# The Vicious Cycle of Biofilm, Host Inflammation and Microvascular Insufficiency in Venous Leg Ulcers

**DOI:** 10.3390/medsci14040453

**Published:** 2026-08-02

**Authors:** Diana Tatarciuc, Irina Mihaela Esanu, Iolanda Foia, Teodor Flaviu Vasilcu, Ana Maria Raluca Pauna, Ilinca Foia, Roxana Ionela Vasluianu, Nicoleta Ioanid, Marius Constantin Moraru, Elena-Roxana Avadanei, Mioara-Florentina Trandafirescu

**Affiliations:** Grigore T. Popa University of Medicine and Pharmacy, 700115 Iasi, Romania; diana.tatarciuc@umfiasi.ro (D.T.); irina.esanu@umfiasi.ro (I.M.E.); iolanda.foia@umfiasi.ro (I.F.); teodor-flaviu.tsr.vasilcu@umfiasi.ro (T.F.V.); paunaanamariaraluca@gmail.com (A.M.R.P.); mg-rom-34346@students.umfiasi.ro (I.F.); nicoleta.ioanid@umfiasi.ro (N.I.); marius.moraru@umfiasi.ro (M.C.M.); elena.avadanei@umfiasi.ro (E.-R.A.); mio.trandafirescu@umfiasi.ro (M.-F.T.)

**Keywords:** venous leg ulcer, biofilm, chronic inflammation, microvascular dysfunction, hyperhomocysteinemia, polymicrobial infection, therapeutic resistance

## Abstract

Chronic venous leg ulcers (VLUs) affect approximately 1% of adults, and up to 30% remain unhealed by 12 months of standard therapy, with rates of recurrence approaching 70%. The chronicity and treatment resistance cannot be explained by the traditional view that venous hypertension alone causes the pathogenesis of VLUs. This narrative review synthesizes evidence from PubMed, Web of Science, and Scopus (2016–April 2026), including original research, systematic reviews, meta-analyses, and clinical trials on the pathophysiology, diagnosis, and treatment of VLU, with a focus on biofilm, inflammation, and microvascular dysfunction. The reviewed studies were critically appraised. The current integrated framework offers a potential mechanistic roadmap for understanding the pathogenesis of VLUs and may help justify integrated therapeutic strategies. This review evaluates the existing evidence, identifies controversies, and highlights areas of knowledge that require further investigation. We analyze emerging data to propose a unified, conceptually distinct framework focused on the reciprocal, self-perpetuating interactions between biofilm, inflammation, and microvascular dysfunction, a triad that may offer a more comprehensive explanation for clinical heterogeneity and therapeutic resistance than venous hypertension alone. Future research should focus on the development of clinically available biofilm diagnostics, rigorous studies of combination therapies and elucidation of molecular links between components of the triad. We suggest that a transition to mechanism-based approaches targeting simultaneously may hold promise for transforming outcomes for millions of people affected by this debilitating condition.

## 1. Introduction

Chronic venous leg ulcers (CLUs), the most advanced manifestation of chronic venous insufficiency, affect up to 1% of adults and account for 70–90% of lower limb ulcers, with substantial impacts on quality of life, depression, and healthcare costs [1,2]. Despite decades of research, their pathophysiology remains incompletely understood. The non-healing phenotype cannot be explained by venous hypertension alone, as many patients with severe venous disease never develop ulcers, while others with mild insufficiency present with wounds that persist for years [3]. This clinical heterogeneity requires a unified biological framework that integrates microbiology, immunology, and vascular biology.

Emerging evidence suggests the possibility of a self-perpetuating triad involving biofilm persistence, dysregulated inflammation, and microvascular insufficiency. Recent data show that formidable biofilm producers constitute 70.8% of infected VLUs, with *Staphylococcus aureus* (94.9%) and *Pseudomonas aeruginosa* (87.5%) predominantly manifesting this phenotype while multidrug-resistant organisms make up 25.8% of all isolates [4]. At the same time, infected VLUs display significantly increased levels of interleukin-6, interleukin-17A, and tumor necrosis factor alpha in wound fluid coupled with hyperhomocysteinemia in 58.9% of cases, a finding that mechanistically connects systemic vascular pathology to local biofilm-associated inflammation [5,6]. These inflammatory mediators stimulate matrix metalloproteinase overexpression and extracellular matrix degradation, perpetuating microvascular injury and creating an environment conducive to biofilm persistence [7].

Although biofilm, inflammation, and microvascular dysfunction have been described individually in chronic wounds, existing models (the fibrin cuff theory, the leukocyte trapping hypothesis, and the biofilm persistence models) treat them as separate processes rather than an integrated cycle [8,9,10]. Existing models generally address these mechanisms separately, rather than conceptualizing their bidirectional interactions as a unified pathogenic system. Our triadic model offers a potential advance by integrating microbiology, immunology, and vascular biology into a single framework; suggesting homocysteine as a possible molecular link between systemic and local pathology; proposing that chronicity may involve cyclical rather than additive interactions; and providing a mechanistic rationale for simultaneous multitarget therapy.

In this narrative review, we synthesize the current evidence supporting the biofilm-inflammation-microvascular triad as an integrative model that explains the self-perpetuating nature of VLU chronicity. We propose that effective management may require a conceptual shift from isolated interventions, compression alone or antimicrobial therapy guided by planktonic susceptibility testing, toward integrated strategies that simultaneously target biofilm eradication, inflammation modulation, and microvascular restoration, a hypothesis that warrants further investigation.

## 2. Materials and Methods

### 2.1. Search Strategy

A comprehensive bibliographic search was performed in 3 major electronic databases: PubMed/MEDLINE, Web of Science and Scopus. The search was focused on studies published in the last decade to capture recent advances in the field. The search strategy combined the following terms and keywords, being strictly correlated with the three core concepts: (1) venous leg ulcers (e.g., “varicose ulcer”, “venous ulcer”, “chronic venous insufficiency”), (2) biofilm and microbiology (e.g., “biofilm”, “polymicrobial”, “*Staphylococcus aureus*”, “*Pseudomonas aeruginosa*”), and (3) host response and microvascular pathology (e.g., “inflammation”, “cytokines”, “homocysteine”, “microcirculation”, “endothelial dysfunction”). Details about the search strategy are presented in the Supplementary Material.

### 2.2. Inclusion and Exclusion Criteria

Articles that addressed the pathophysiology, diagnosis, or treatment of venous leg ulcers, biofilm formation, host inflammatory response, or microvascular dysfunction in venous leg ulcers (VLU) were included. Original research, systematic reviews, meta-analyses, or clinical trials published in English between 2016 and April 2026 were included in this narrative review. Studies that focused exclusively on other wound types (e.g., diabetic foot ulcers, pressure ulcers) with no relevance to VLUs, animal studies, case reports, and conference abstracts were excluded. Studies on therapy were included if they specifically addressed the management of VLUs.

## 3. Biofilm Component and Microbial Persistence

### 3.1. The Biofilm in Chronic Wounds

Chronic wound biofilms are complex microbial consortia encapsulated in a self-produced extracellular polymeric substance (EPS) matrix [11,12,13,14,15]. The matrix constitutes an active and versatile microenvironment that significantly influences bacterial physiology and host–pathogen interactions [16,17,18,19].

In 2021, Versey et al. reported that biofilms provide bacteria with numerous survival advantages. These include protection from phagocytosis by neutrophils and macrophages, sequestration of divalent cations required for complement activation, and a 1000-fold reduction in the penetration of antibiotics and antiseptics compared with planktonic bacteria [17]. In addition, by acting as a reservoir for extracellular enzymes that degrade host tissue constituents such as collagen, elastin, and fibrin, the matrix promotes wound chronicity by supporting tissue degradation [19].

Both bacterial lysis and extracellular trapping of host neutrophils (NETs) produce extracellular DNA in the matrix, which increases inflammation and acts as a structural framework to prevent mechanical disintegration of the biofilm [17]. Biofilms in chronic wounds persist even after adequate debridement and systemic antibiotic therapy, due to their complex structural organization, requiring therapies that disrupt the EPS matrix and target its protected, metabolically distinct subpopulations [18,19,20,21,22].

There are significant ramifications for the treatment of VLUs. For instance, because the biofilm phenotype renders the bacterium operationally resistant, a wound swab displaying a “susceptible” organism may give false comfort [19,23].

In 2026, Molasy and Wrzosek highlighted that microbial communities persist through a dynamic, four-step process known as the biofilm life cycle [19]. Planktonic bacteria, frequently introduced through skin contamination or epithelial barrier disruption, attach to the exposed wound surface [24]. This attachment is mediated by reversible physicochemical interactions, which are rendered irreversible by the production of bacterial adhesins.

Once firmly anchored, these bacteria multiply and begin to secrete extracellular polymeric material. Eventually, they develop into microcolonies, where they aggregate into small clusters covered by a matrix that protects them from systemic antibiotics and host immune cells. As the biofilm develops, the microcolonies grow into complex three-dimensional structures with a sophisticated network of water channels that facilitate waste removal and nutrient exchange. Quorum sensing coordinates the production of virulence factors and initiates stress responses that enhance antimicrobial tolerance [17,19]. Environmental stress, waste product accumulation, or nutritional deprivation activate the dispersal phase. This stimulation triggers the release of unattached bacteria or bacterial aggregates, which may initiate new infection sites within the wound or elsewhere [19].

The contamination, in its extreme form, is defined as the presence of non-replicating microorganisms on the wound surface without tissue penetration or host response, which is of no clinical significance [19]. The transition to colonization occurs when bacteria begin to replicate and adhere to the wound bed but remain confined to the superficial layers and do not trigger any host inflammatory response. At this point, healing can proceed normally. Several studies demonstrate that colonization is a critical point when the bacterial load is usually >10^5^ colony-forming units/g tissue [23,25]. This phase frequently aligns with biofilm formation, where bacteria transition from a planktonic to a sessile state. In this state, they secrete a protective extracellular polymeric substance that shields them from host immunity, antimicrobials, and environmental stressors [17,19].

### 3.2. Microbial Synergy and Persistence of Pathogens in VLUs

Polymicrobial biofilms appear to be a feature of many chronic VLUs, which may not be a consequence of a single pathogenic species but instead may involve complex, synergistic interactions between species that could fundamentally alter the course of infection and therapy [18,19]. Polymicrobial biofilms in VLUs are predominantly driven by *Staphylococcus aureus* and *Pseudomonas aeruginosa* [19,24].

**Figure 1:**
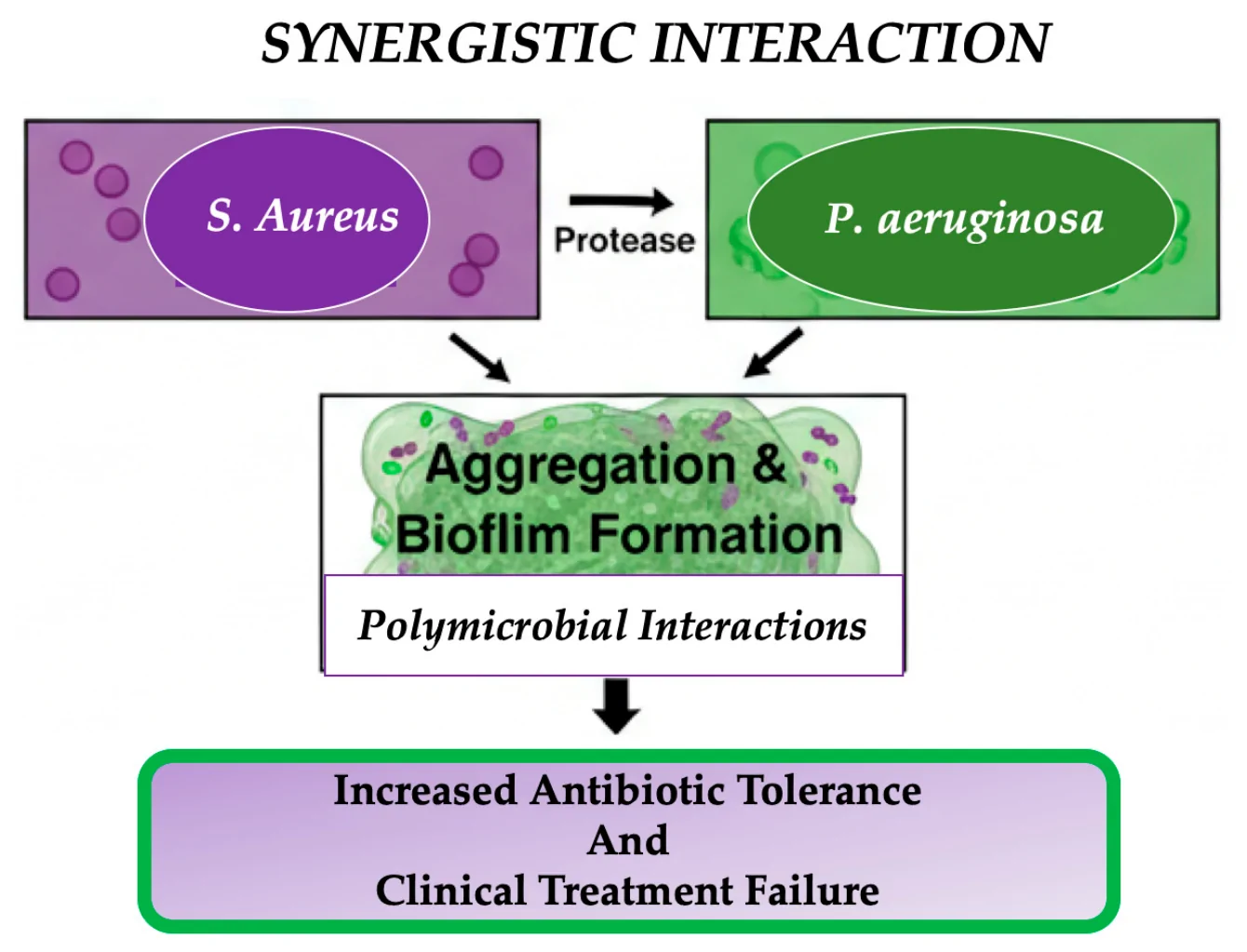
Polymicrobial biofilm ecosystem in VLUs.

Some researchers claim that co-infection with these two pathogens has been shown to be more virulent, persistent and associated with poor response to therapy than monospecies infections [19,24]. This cooperation relies on particular molecular interactions, such as *P. aeruginosa* secreting proteases which are able to activate aggregation pathways in *S. aureus*, therefore paradoxically assisting in the formation and persistence of its biofilm. Furthermore, these polymicrobial interactions may increase tolerance to antimicrobial treatments. This tolerance occurs because thicker and more complex mixed biofilms and reduced bacterial metabolic activity protect the community from therapies that would otherwise be effective [17,19]. The synergistic interactions and the enhanced therapeutic failure that follows may be major factors contributing to the chronicity of VLUs, potentially transforming the wound into a resistant and hard-to-erase microbial consortium (Figure 1) [18,20]. In 2024 and 2025, Kim et al. observed that biofilm formation is a context-dependent trait, influenced by host factors such as immune status, comorbidities, and the wound microenvironment [25,26]. This finding underscores the importance of a diagnostic and therapeutic approach that goes beyond pathogen identification and emphasizes the need to understand host–microbe interactions (Figure 1).

### 3.3. Biofilm-Mediated Antimicrobial Resistance and Immune Evasion in VLUs

Several studies demonstrated that moving from planktonic to biofilm-associated growth influences the behavior of bacteria with respect to antimicrobial agents and the host immune system [17,18]. This striking resistance is the product of a combination of structural, physiological and genetic mechanisms that convert the biofilm into a structurally protected bacterial population against therapeutic and immunological attacks [17,19].

Positively charged antibiotics like aminoglycosides may bind to the negatively charged structures of this matrix, reducing their capacity to reach targeted bacteria. In addition to this barrier effect, the architecture of the biofilm generates microenvironments with differences in the levels of nutrients and oxygen, resulting in metabolic heterogeneity between different bacterial subpopulations [17,19]. Thus, cells located in deep hypoxic zones have a reduced metabolism and slow division, becoming less vulnerable to antibiotics that act on actively proliferating cells, such as beta-lactams [17,27]. Some authors have reported that the proximity of biofilm cells also favors horizontal gene transfer. This process allows the exchange of mobile elements (plasmids, integrons, transposons) carrying resistance genes, thus accelerating the adaptation and spread of resistance within the community. These heritable mechanisms contribute to phenotypic tolerance, complicating therapeutic options (Table 1) [18,19,24].

Immune cells such as neutrophils and macrophages are not exposed to bacterial surface but to parts of the EPS matrix, thus hiding pathogens efficiently and impairing phagocytosis. Biofilm formation has been shown to affect complement deposition, for example by reducing C-reactive protein and complement component C1q binding, thereby reducing activation of the classical complement pathway. Neutrophil dysfunction is also a feature of the host response to biofilms. Neutrophils that migrate to the site of infection and interact with the biofilm lose their phagocytic efficiency and become susceptible to bacterial virulence factors. This chronic inflammatory response, with persistent neutrophil activation, oxidative stress and proteolytic enzyme release, leads to tissue damage and further impairs infection resolution. Interestingly, host immune cells such as extracellular DNA can be incorporated into the EPS matrix, paradoxically stabilizing the biofilm structure and increasing its resistance [17,25,26].

The mechanisms described above have important clinical implications, such as that standard antibiotic susceptibility tests, which are based on planktonic cultures, do not reflect the actual susceptibility of bacteria embedded in biofilms. The EPS matrix, decreased metabolic activity and enhanced antibiotic tolerance require a much higher antibiotic concentration than usually achieved in vivo to achieve a bactericidal effect [17,18,19]. This clinical reality suggests that a conceptual shift toward biofilm-targeted diagnostic and therapeutic strategies may be warranted, although prospective studies are needed to confirm their superiority over conventional approaches [17,18,19,20,21,22,28].

### 3.4. The Main Pathogenic Debate on Biofilm vs. Planktonic State

A major controversy in the microbiology of VLUs is whether biofilm is the universal pathogenic state or whether it is limited to a subset of cases. This has significant therapeutic relevance, as biofilm-associated bacteria are 1000-fold more tolerant to antibiotics and require completely different therapeutic strategies [17].

Molecular studies (FISH, confocal microscopy) demonstrate biofilm structures in 70–80% of chronic wound specimens, compared with <10% in acute wounds. Deep tissue biopsies reveal polymicrobial biofilms in up to 85% of non-healing VLUs [19]. The biofilm phenotype may help explain why standard antibiotic therapy, guided by planktonic susceptibility testing, frequently fails [17,20]. Proponents argue that biofilms are universally present but remain undetected because standard culture identifies bacteria embedded in the biofilm in only 30% of cases (the VBNC state).

Alternative evidence suggests that biofilm is variable, not universal. Some prospective cohorts report a biofilm prevalence of only 40–60%, indicating that many chronic VLUs heal without biofilm-directed therapy [11,18]. The wound may be chronic due to persistent venous hypertension, non-healing inflammation, or host comorbidities unrelated to the biofilm [29,30,31]. Also, the presence of biofilm is not always associated with clinical infection. Many wounds with biofilm do not show signs of infection [25]. This difference is due to methodological heterogeneity, such as underestimation of biofilm by standard cultures (detecting only planktonic organisms), and molecular techniques can detect non-viable DNA [20].

Detection of biofilm in non-healing VLUs in most studies (70–80%) uses multiple detection modalities, but rarely as the sole determinant of chronicity [17,19]. Available evidence appears to support a continuum model. Biofilm is a facultative adaptive response to the hostile wound microenvironment (hypoxia, oxidative stress, immune pressure), as opposed to a binary switch [17,31]. The transition depends on ulcer duration (>12 months increases the prevalence from 60% to 85%), antibiotic exposure (selects for biofilm-forming strains), and host immune status [25,26,32]. This is why some patients respond to debridement alone (immature biofilm), while others require multimodal therapy (mature biofilm).

The biofilm-centric perspective supports routine biofilm-directed diagnostics/therapies in all chronic VLUs. The subset perspective supports selective application. The continuum model recommends risk-stratified approaches, such that patients with high-risk characteristics (>12 months duration, prior antibiotics, exudate/malodor) require aggressive biofilm diagnostics/therapy, while other patients may initially receive standard care with early escalation if healing is blocked [25,26,32].

## 4. The Immune Microenvironment of VLUs

### 4.1. Chronic Inflammation as a Driver of Non-Healing

Although inflammation is essential for normal wound healing, its persistence in VLUs creates a pathological state that prevents tissue repair. Normal healing involves inflammation, a transient and tightly regulated response that clears debris and signals the arrival of repair cells. The physiological inflammatory response leads to bad outcomes in chronic wounds. In VLUs, however, this process goes awry. The wound gets stuck in a pathological state of chronic inflammation and fails to transition from the inflammatory to the proliferative phase [25,26]. This arrest defines the chronic wound and leads to the relentless tissue destruction that characterizes VLUs.

Physiological wound healing consists of four overlapping and highly regulated phases, such as hemostasis, inflammation, proliferation and remodeling. The inflammatory phase is characterized by leukocyte recruitment, clearance of pathogens and debris and release of growth factors that will initiate repair following injury. Once the wound is cleansed, macrophages switch from a pro-inflammatory (M1) to a pro-reparative (M2) phenotype, orchestrating the transition to the proliferative phase. In this phase, new tissue is formed through angiogenesis, granulation, and re-epithelialization. The process culminates in remodeling, in which the extracellular matrix is reorganized to restore tissue strength [17].

VLUs appear to be characterized by a prolonged and sustained inflammatory phase that may prevent dermal and epidermal cells from responding to healing signals [25,26]. Instead of resolving, inflammation persists through the uncontrolled recruitment and activation of proinflammatory cells. Macrophage dysfunction appears to play a central role. The normal transition from M1 to M2 is prevented, leading to a build-up of pro-inflammatory M1 macrophages which promote tissue damage. These cells secrete high levels of chemokines, pro-inflammatory cytokines (IL-6, IL-17A, and TNF-α) and reactive oxygen species (ROS) in a constitutive manner, creating a hostile microenvironment for regeneration [17]. This is complicated by neutrophil dysfunction, with increased neutrophil activity and the formation of neutrophil extracellular traps (NETs), further increasing the inflammatory mediators and proteases.

The hallmark of chronic venous insufficiency is venous hypertension that initiates the pathological cascade. Elevated venous pressure in the lower extremities sequesters excess leukocytes in the microvasculature. Activated leukocytes stick to the capillary endothelium and release proteolytic enzymes, free radicals and inflammatory mediators, damaging the capillary wall, increasing permeability and causing local tissue injury [17,33].

Early theories proposed that venous hypertension leads to the deposition of peri-capillary “fibrin cuffs,” layers of fibrinogen and other macromolecules that act as physical barriers, impairing oxygen diffusion and nutrient delivery to the skin. The picture is much more complex than simple hypooxygenation. The presence of perivascular leukocyte infiltration alongside fibrin cuffs suggests an active, dynamic inflammatory process, rather than the formation of a passive diffusion barrier [17]. In the microenvironment of the chronic wound, inflammation is not a transient response, but a persistent pathological state that actively impairs healing [17,25,26].

**Figure 2:**
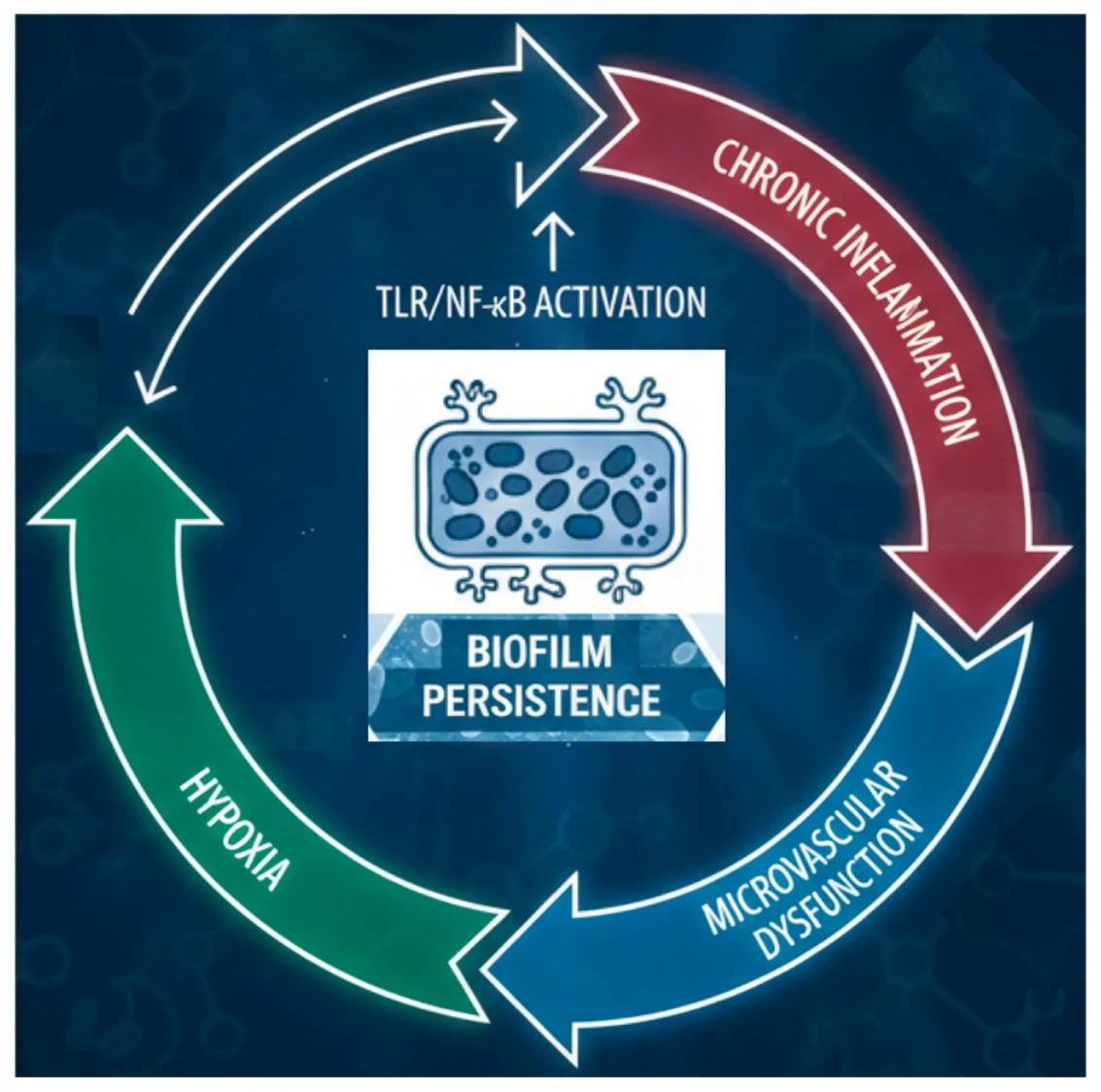
The cyclical interactions: molecular interactions that determine chronicity.

The mechanism that may keep the lesion in a state of constant inflammation and impair healing has been described as a potential vicious cycle (Figure 2).

This is a circular loop that converts a theoretically repairable tissue defect into a difficult-to-resolve clinical problem. Cellular senescence, a state of irreversible growth arrest induced by prolonged inflammation and oxidative stress, is the starting point of the cycle for chronically active fibroblasts and endothelial cells. Instead of remaining dormant, these senescent cells adopt a highly active secretory phenotype, termed the senescence-associated secretory phenotype (SASP). Through SASP, they release a toxic mixture of chemokines, matrix-degrading proteases, and pro-inflammatory cytokines into the wound environment. This release of SASP not only reflects cellular aging, but also stimulates and activates inflammation, attracting and activating neutrophils and macrophages that, instead of healing the wound, perpetuate tissue damage [17,25,26].

The relentless inflammatory environment, in turn, increases oxidative stress. Reactive oxygen species (ROS) from dysfunctional neutrophils and macrophages flood the wound bed, causing oxidative damage to lipids, proteins, and DNA. The inflammatory response is amplified by this oxidative attack, resulting in a feed-forward cycle that overwhelms the body’s natural antioxidant defenses. The resulting tissue damage extends beyond the cellular compartment to the extracellular matrix, where the balance between production and degradation is catastrophically disrupted [17,25,26]. Persistent elevation of matrix metalloproteinases (MMPs) is essential for this disruption.

Chronic inflammation induces an increase in MMP-2, MMP-8, and MMP-9, while simultaneously reducing their natural inhibitors, tissue inhibitors of metalloproteinases (TIMPs) [34]. The resulting protease imbalance causes destructive degradation of the extracellular matrix (ECM), changing the wound environment from a positive state to one that promotes healing. Collagen, elastin, and fibronectin are broken down faster than they can be synthesized, eroding the structural framework necessary for cell migration, granulation tissue development, and reepithelialization [17,34]. As the ECM disintegrates, the wound bed loses its potential to support the formation of new blood vessels, resulting in decreased angiogenesis. Vascular endothelial growth factor (VEGF) is upregulated in response to hypoxic injury, but the angiogenesis it stimulates is dysregulated and produces fragile, leaky capillaries that do not restore functional activity [29,35]. Instead of delivering oxygen and nutrients, these dysfunctional veins prolong hypoxia, the very condition that led to their formation. Cellular senescence is hastened, and the cycle is repeated with oxygen deprivation closing the loop. This cycle explains why cells remain trapped in a chronic, non-healing state that cannot be resolved by conventional wound treatment, not transitioning from the inflammatory to the proliferative phase [17,29,30].

### 4.2. The Host–Microbiome Interface: A Bidirectional Relationship

The host–microbe relationship in VLUs is not a unidirectional interaction, but rather a dynamic, bidirectional relation. In this relation, the host’s immune status shapes microbial behavior, while microbial virulence factors reciprocally modulate host inflammatory responses. The reciprocity may be a key determinant of whether a wound heals or becomes chronic. Host factors are the major modulators of susceptibility to biofilm-associated infections and clinical outcomes [17,25,26]. Age > 65 years, ulcer duration > 12 months, wound surface area > 8.25 cm^2^, and comorbidities including hypertension, diabetes, ischemic cardiomyopathy and history of deep vein thrombosis are independent risk factors for infection and delayed healing [25,26,32]. Systemic immunosuppression, such as that seen in HIV, corticosteroid therapy, or organ transplantation, further limits the host’s ability to contain microbial invasion. The local immune system is profoundly dysfunctional in the wound environment.

These local and systemic immune defects provide a permissive environment for biofilm-associated bacteria to establish a persistent infection. On the other hand, bacteria are not passive recipients of impaired host immunity. They actively manipulate the inflammatory milieu of the host with sophisticated virulence strategies. This nuance is very well captured by the concept of “contextual pathogenicity”, where bacterial virulence is not an absolute property, but depends on the context and is influenced by the host microenvironment. Under homeostatic conditions, commensal microbes can promote epithelial and immune homeostasis. However, when host defense mechanisms are compromised through barrier disruption, immunosuppression, or chronic inflammation, the same organisms can adopt pathogenic behavior. An example of the range of pathogenicity in context is seen in VLUs where *Staphylococcus epidermidis*, a commensal skin colonizer, can be selected for antimicrobial resistance and biofilm formation in the chronic wound microenvironment and impair reepithelialization through biofilm-dependent induction of proinflammatory cytokines [17,35].

A particularly innovative approach to this bidirectional relationship is emerging from recent studies of *P. aeruginosa* in VLUs. The explanation for this paradox lies in the active suppression of host immunity by the bacterium, exemplified by *P. aeruginosa* elastase B (LasB). Indeed, LasB degrades a wide range of proinflammatory cytokines (e.g., G-CSF, GM-CSF, IFN-γ, IL-1ra, IL-6, IL-12p40, IL-23, and TNF-α) and chemokines (e.g., Gro-α, IL-8, IP-10, MCP-1, MIP-1α, and MIP-1β) in the extracellular environment, thereby effectively dismantling the host inflammatory signaling network [31]. This active immunomodulation does not affect intracellular cytokine production or transcription factor activation, a sophisticated strategy that allows the bacterium to persist with the host aware of the threat. This bidirectional relationship has profound clinical implications. Standard antibiotic therapy based on planktonic susceptibility testing is not effective because it does not consider the biofilm phenotype or active immunomodulatory strategies of pathogens such as *P. aeruginosa* [17].

There is a significant knowledge gap and few studies have systematically correlated virulence profiles of VLU isolates with host immune responses or clinical outcomes [17,36]. However, recent evidence suggests that the chronic wound microenvironment itself may select for more virulent and resistant strains, as evidenced by the functional selection of antibiotic-resistant, biofilm-forming *S. epidermidis* within VLUs. Understanding the molecular mechanisms that drive this bidirectional host–microbiome interface is essential for the development of next-generation therapeutic strategies. Such strategies must simultaneously target bacterial virulence, restore immune function, and interrupt the cycle of chronicity (Table 2).

### 4.3. Molecular Mechanisms of Oxidative Stress and Mitochondrial Dysfunction

The chronic VLU microenvironment favors sustained production of ROS from various sources. O_2−_ reacts with NO to produce peroxynitrite (ONOO^−^), a potent nitrating agent. H2O2 produces the highly reactive hydroxyl radical (•OH) via Fenton chemistry. Endogenous antioxidants, such as glutathione (GSH), SOD, catalase, and glutathione peroxidase, are overtaxed for several reasons, namely (a) NOX-induced NADPH depletion; (b) oxidative inactivation of SOD/catalase; (c) impaired synthesis under hypoxic conditions. GSH/GSSG ratios decrease to ≤5:1 in VLU fluid versus >10:1 in healing wounds [17,31].

ROS cause injury through three pathways:

- Lipid peroxidation: •OH abstracts hydrogen from polyunsaturated fatty acids, generating MDA (≥2.5 μM in VLU fluid vs. ≤0.8 μM in acute wounds), inversely correlated with healing at 12 weeks [17].
- Protein carbonylation: ROS oxidize arginine, lysine, proline, and threonine, introducing carbonyl groups, leading to inactivation of TIMPs, exacerbating protease imbalance. Carbonyl content ≥ 5 nmol/mg protein predicts failure to achieve ≥40% wound reduction at 4 weeks [17,34].
- DNA damage: ROS induce 8-OHdG (≥15 ng/mL in VLU fluid), correlating with ulcer duration and poor healing [17].

Mitochondria are particularly vulnerable, as they are the main endogenous sources of ROS, via ETC complexes I and III. mtDNA is not protected by histones, has limited repair capacity, and is located in close proximity to the ETC. ROS-induced mtDNA deletions/mutations, particularly in D-loop genes and ETC subunits, impair ETC function, leading to increased electron leakage and ROS generation in a feed-forward cycle.

In summary, these mechanisms include ATP depletion (40–60% of normal levels in VLU fibroblasts), mPTP opening with cytochrome c release, and intrinsic apoptosis through caspase-9/3 activation. Biomarkers (MDA, protein carbonyls, 8-OHdG) may identify some of the necessary elements for emerging antioxidant therapies (MitoQ, N-acetylcysteine, ROS-scavenging nanoparticles, NOX inhibitors), although clinical validation remains limited [17,31,37].

### 4.4. Molecular Mechanisms of NLRP3 Inflammasome-Mediated Inflammation

Inflammasomes are multiprotein complexes that activate caspase-1, thereby promoting the maturation and secretion of IL-1β and IL-18. The NLRP3 inflammasome, which comprises the NLRP3 sensor, the ASC adaptor, and procaspase-1, is most relevant to chronic inflammation and is activated by diverse microbial and endogenous signals in the VLU microenvironment [17,31,33].

NLRP3 activation requires two sequential signals:

- Signal 1 (Priming): TLR engagement (via bacterial LPS, lipoteichoic acid, or DAMPs such as HMGB1) activates NF-κB, upregulating the transcription of NLRP3, pro-IL-1β, and pro-IL-18;
- Signal 2 (Activation): Several triggers induce NLRP3 oligomerization and caspase-1 cleavage, including: (a) K^+^ efflux via pore-forming toxins (α-hemolysin from *S. aureus*) and ATP-gated P2X7 receptors; (b) lysosomal disruption by uric acid crystals; and (c) release of mitochondrial ROS and mtDNA.

Following signal 2, NLRP3 oligomerizes, recruits ASC to the particle, and activates caspase-1. Active caspase-1 cleaves pro-IL-1β and pro-IL-18 to their mature forms and cleaves gasdermin D, inducing pyroptosis, a potent inflammatory cell death that releases DAMPs, amplifying inflammation. *P. aeruginosa* uses several strategies to activate NLRP3 in VLUs:

- Priming signals: LPS (TLR4), flagellin (TLR5), and the porin OprF (TLR2) activate NF-κB;
- Activating signals: The type III secretion system delivers ExoU (phospholipase A2) and ExoY, disrupting membranes and causing K^+^ efflux. Pyocyanin induces mitochondrial ROS production and mtDNA release.

Wound fluid IL-1β levels in VLUs range from 15–45 pg/mL, compared with <5 pg/mL in acute wound healing. Levels ≥ 15 pg/mL predict failure to achieve ≥40% wound area reduction at 4 weeks (PPV: 73%, 95% CI: 61–83%). IL-18 levels ≥ 50 pg/mL are associated with ulcer duration > 12 months and increased risk of infection.

Partial, rather than complete, inhibition of NLRP3 may preserve antimicrobial functions while attenuating pathological inflammation. Biomarker-guided therapy, such as selection of patients with IL-1β ≥ 15 pg/mL, could optimize the benefit-risk ratios [17,31,33].

### 4.5. Pseudomonas aeruginosa in VLU: Pathogen or Opportunist?

The role of *P. aeruginosa* in VLU is still a matter of debate: whether it is a pathogen that actively promotes pathogenesis or an opportunist that colonizes a compromised wound environment. There is evidence for both positions. Evidence that VLU colonized with this pathogen agent has significantly poorer outcomes, such as larger wound surface area (3–4 times larger), delayed healing, and increased chronicity compared with uncolonized ulcers, suggests an active contribution to disease progression [17,24].

The organism has an extensive arsenal of virulence: the type III secretion system injects ExoS, ExoT, ExoU and ExoY directly into host cells, disrupting epithelial integrity and repair mechanisms; the type II secretion system secretes elastases (LasA, LasB) and proteases that degrade extracellular matrix and complement components; and pyocyanin, a redox-active toxin, induces oxidative stress and damage to host cells. Quorum sensing allows for the coordination of virulence expression across the population, allowing synchronized invasion of tissues [31]. *P. aeruginosa* actively subverts host immunity using a variety of mechanisms. The LasB protease cleaves proinflammatory cytokines such as IL-6, TNF-α and IFN-γ, preventing bacterial clearance by these cells, while inducing NLRP3 inflammasome-mediated production of IL-1β, causing tissue damage while escaping immune clearance.

The organism also cleaves complement components and exhibits resistance to antimicrobial peptides, thereby surviving host defenses. In addition, *P. aeruginosa* forms biofilms in at least 60% of chronic wounds compared with only 6% of acute wounds, providing 100- to 1000-fold greater antimicrobial resistance and protection from phagocytosis [17,19]. The bacterium is also able to migrate deep into tissue, embedding itself in the extracellular matrix, making eradication with antibiotics highly unlikely.

Comparative studies show that *P. aeruginosa* strains isolated from chronic wounds are generally less virulent than strains from bloodstream infections. Isolates from chronic wounds demonstrate significantly reduced motility by rotation and spasms, lower proteolytic activity, and predominantly moderate (rather than strong) biofilm formation compared with acutely infected strains. Pyocyanin production also appears to be lower, although this is not always statistically significant. This trend suggests a genetic adaptation, with virulence factors being selected over time, similar to cystic fibrosis, where the virulence is progressively attenuated during chronic infection [11,18]. The presence of this pathogen is strongly associated with host factors: ulcers > 10 cm^2^ and/or present for >12 months have higher colonization rates [25,26,32]. The chronic inflammatory state itself, rather than bacterial virulence, may be the main determinant of persistent ulceration. *P. aeruginosa* uses anaerobic nitrate respiration as a survival strategy in oxygen-deficient wounds, an adaptation for persistence rather than an active pathogenic mechanism. Furthermore, *P. aeruginosa* is a member of a polymicrobial complex and no single organism can induce chronicity; rather, the interaction between species may be competitive, thus reducing the role of any one pathogen [29,30,31].

*P. aeruginosa* is an opportunistic pathogen, not inherently pathogenic to healthy tissue, but pathogenic in the context of a host with compromised defenses. The wound environment provides permissive conditions (hypoxia, chronic inflammation, immune dysfunction) that allow colonization. Once established, the organism actively contributes to chronicity through biofilm formation, virulence expression, and immune manipulation, resulting in a self-perpetuating cycle. This dual nature reconciles apparent contradictions, such as strains demonstrating low virulence compared with isolates from acute infections (supporting an opportunistic role) but correlating with poor clinical outcomes (supporting a pathogenic role). Thus, the distinction between “colonization” and “infection” is context-dependent and determined by host susceptibility, bacterial genotype, and the wound microenvironment [25,29,30,31].

## 5. Microvascular Failure as a Vascular Consequence of Chronic Inflammation

### 5.1. The Pathophysiology of Venous Ulceration

The pathophysiological cascade of VLUs begins with venous hypertension, the hemodynamic hallmark of chronic venous insufficiency. Valvular insufficiency, venous obstruction, or failure of the calf muscle pump leads to increased venous pressure in the lower extremities. This increased pressure initiates a series of microvascular events that transform mechanical injury into a biological cascade of inflammation and tissue destruction. Long-standing venous hypertension transmits the increased hydrostatic pressure to the cutaneous microcirculation with dilation of capillary walls and widening of interendothelial spaces. Structural damage leads to loss of capillary barrier integrity, leading to increased vascular permeability and extravasation of plasma proteins, including fibrinogen, into the interstitial space [29,30].

In 2023, the molecular basis of endothelial dysfunction in chronic venous disease was reviewed in detail by Costa et al., who highlighted significant pathways, including upregulation of adhesion molecules (ICAM-1, VCAM-1), endothelial dysfunction of nitric oxide synthase, activation of the NF-kB inflammatory cascade, and epigenetic changes affecting endothelial gene expression [18]. These molecular changes, combined with the mechanical effects of venous hypertension, result in a proinflammatory, prothrombotic endothelial phenotype that is prone to leukocyte adhesion, capillary leakage, and tissue injury. In particular, the authors emphasize that endothelial dysfunction is not a passive consequence of venous hypertension. It represents an active, progressive process involving persistent transcriptional changes that perpetuate vascular pathology even after hemodynamic correction, a finding that supports our triadic model’s emphasis on self-perpetuating mechanisms [31].

The pathophysiology of venous ulceration is based on the central tenet of the “leukocyte trapping” hypothesis, which suggests that leukocytes become trapped in the lower limb microcirculation in states of venous hypertension. In normal physiology, leukocytes flow through capillaries with minimal or no impedance. However, an increase in venous pressure causes a reduction in the perfusion pressure gradient, resulting in slower capillary blood flow, increased leukocyte transit time, and increased adhesion to activated endothelium [17,29]. Adherent leukocytes further obstruct the capillary lumen, establishing a self-perpetuating cycle of reduced flow, increased trapping, and localized ischemia.

Leukocytes, mainly neutrophils and macrophages, are activated by the hypoxic and inflammatory microenvironment once trapped in the microcirculation. This activation results in the secretion of a cascade of proteolytic enzymes, such as matrix metalloproteinases (MMPs) and elastase, reactive oxygen species (ROS), and a spectrum of proinflammatory cytokines, such as IL-6, IL-17A, and TNF-α [30,33,34]. This release of tissue-damaging molecules diffuses into the surrounding interstitium and leads to endothelial damage, extracellular matrix degradation, and further amplification of the inflammatory response. The balance between MMPs and their endogenous inhibitors (TIMPs) is disrupted, resulting in increased proteolysis during matrix deposition and failure of the wound to develop functional granulation tissue. This persistent leukocyte activation may represent a biological link between venous hypertension and tissue destruction, and the duration of this process may be a signature of non-healing VLU [17,30,34].

Increased capillary permeability leads to extravasation of fibrinogen and other plasma proteins into the dermal interstitium. Fibrinogen is activated in the inflammatory environment and polymerizes to form pericapillary fibrin clots, a typical histopathological finding of venous ulcers and lipodermatosclerosis. These clots were initially suggested to be a physical barrier to diffusion, but they may restrict the delivery of oxygen, nutrients, and growth factors to the overlying epidermis. However, current evidence suggests that the fibrin clot may also trap growth factors and cytokines, preventing them from reaching target cells and further impairing wound healing [17,29,35].

The fibrin clot is now appreciated as an active component of the inflammatory microenvironment, not just a passive barrier to diffusion, sequestering growth factors, promoting fibroblast proliferation and perpetuating fibrosis. The most characteristic histopathological feature of the venous ulcer microenvironment may be the presence of proliferative, tortuous capillaries which form “capillary tufts” or “capillary loops” in the papillary dermis. This maladaptive angiogenesis is the skin’s unsuccessful compensatory response to chronic hypoxia and tissue injury. Unlike the orderly and hierarchical angiogenesis of normal wound healing, this aberrant vascular proliferation results in functionally compromised vessels with leaky endothelium, irregular lumens and diminished perfusion capacity.

These fragile capillaries are susceptible to rupture, leading to hemosiderin deposition and secondary tissue injury. The tortuosity and irregular architecture are the consequence of pathological angiogenesis induced by the sustained high levels of VEGF in the inflammatory microenvironment. Paradoxically, this does not improve the tissue oxygenation but perpetuates the inflamed and permeable state of the wound bed. This structural change may be a hallmark of advanced venous insufficiency and a potential marker of microvascular insufficiency that could lock the VLU into a state of chronic non-healing.

**Figure 3:**
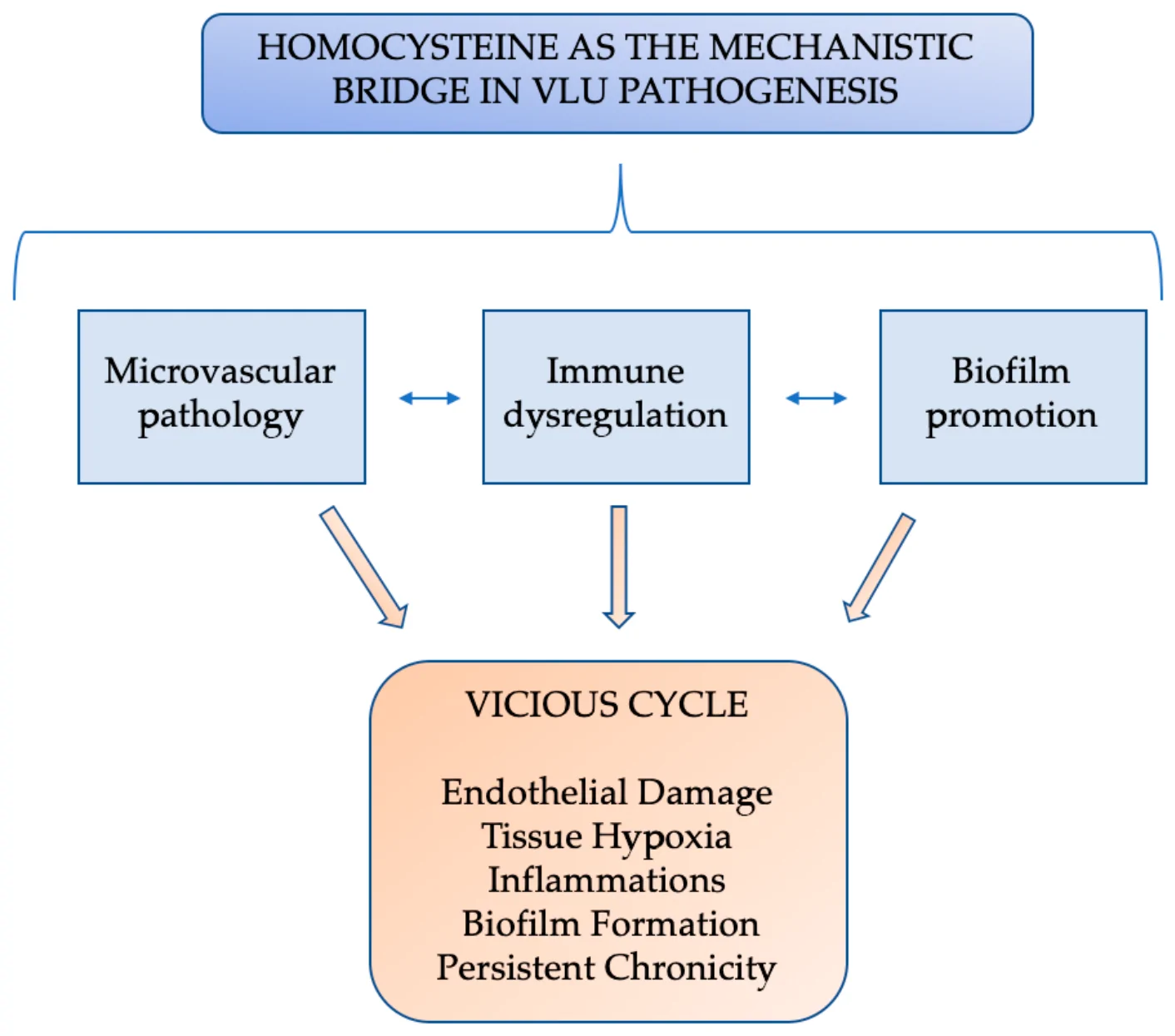
The framework of vicious cycle of vascular pathology.

Homocysteine (Hcy) is a modifiable risk factor for VLU. Hyperhomocysteinemia causes endothelial damage, favors biofilm formation and is associated with risk of infection and poor healing [33,36]. Clinically, Hcy levels > 15 µmol/L identify patients at high risk and may guide B vitamin supplementation [33]. Wound fluid biomarkers (IL-6, TNF-α, MMPs) further refine prognosis [34,36]. The clinical relevance of this framework is exemplified by the observation that homocysteine is significantly more prevalent in infected VLUs than in uninfected ones. This finding supports the idea that homocysteine may function both as a biomarker of infection risk and as a mechanistic factor in the infection-chronicity cycle (Figure 3).

### 5.2. Molecular Mechanisms of Microvascular Dysfunction in VLUs

In VLU, microvascular insufficiency may result from progressive structural and functional changes in the endothelium, beyond capillary damage caused by venous hypertension [29,30,31]. The glycocalyx is a 0.5–2.0 µm layer of heparan sulfate, chondroitin sulfate, hyaluronan, and glycoproteins that maintains barrier function, mediates leukocyte adhesion, and transduces shear stress. In VLUs, MMP-2/-9, heparanase, and TNF-α (via ADAM17) degrade this layer [17,31,34]. Effects include increased permeability, leukocyte hyperadhesion, and impaired mechanotransduction. Serum syndecan-1 ≥ 50 ng/mL is associated with impaired healing [29,31]. Chronic inflammation causes changes in endothelial metabolism, shifting from fatty acid oxidation to glycolysis, a Warburg-like effect regulated by HIF-1α and NF-κB.

Reduced ATP production (36 to 2 ATP/glucose)—Glycolytic intermediates (e.g., fructose-1,6-bisphosphate) stimulate inflammatory pathways. ER stress (BiP/GRP78, upregulation of CHOP) and impaired mitochondrial function lead to increased endothelial apoptosis and senescence, resulting in barrier dysfunction and impaired angiogenesis. The adhesion cascade is a stepwise process:

- Unfolding—E/P-selectins induced by TNF-α/IL-1β;
- Activation—IL-8, MCP-1 (chemokines);
- Firm adhesion—binding of integrins (ICAM-1/VCAM-1) stimulated by IL-6/TNF-α;
- Transmigration—across endothelial junctions [17,29,31].

Venous hypertension leads to prolonged leukocyte transit, increased adhesion, and subsequent tissue damage. Ang-1 secreted by pericytes activates Tie2 to stabilize the vessel, and Ang-2 is secreted during inflammation to destabilize the vessel. The increased Ang-2/Ang-1 ratio in VLUs leads to maladaptive angiogenesis with leaky and tortuous vessels that fail to restore perfusion. VEGF also phosphorylates VE-cadherin, thereby increasing vessel permeability, which perpetuates edema and tissue hypoxia [29,31,35].

Vascular remodeling, intimal hyperplasia, medial fibrosis, and collagen deposition cause progressive lumen narrowing, which impedes vasodilation and nutrient delivery, further perpetuating hypoxia. Venous hypertension leads to inflammation and leukocyte recruitment, which leads to glycocalyx degradation, adhesion, and metabolic disturbances. Dysregulation of the Ang/Tie2 and VEGF pathways results in leaky, nonperfused vessels, and senescence and remodeling progressively impair microvascular function. This cycle of hypoxia and inflammation may perpetuate the non-healing phenotype of VLUs [17,29,30,31].

## 6. Clinical Translation of the Biofilm–Inflammation–Microvascular Framework

The molecular framework described by Costa et al. offers potential biomarkers for monitoring disease progression and response to treatment [18]. Specifically, the adhesion molecule and cytokine profiles they describe may serve as surrogates for the severity of endothelial dysfunction. In the context of our triadic model, changes in these molecular markers could reflect the impact of biofilm-directed therapy on endothelial function or the efficacy of anti-inflammatory interventions in restoring vascular homeostasis. This supports the potential of a biomarker-based approach for personalized management of VLUs, although this remains at the investigational stage [31].

The proposed unified biofilm-inflammation-microvascular construct, if validated, would mandate a paradigm shift in VLUs diagnosis and management. Current diagnostic tools were not designed to detect biofilm-related infections, and therapeutic approaches remain fragmented, with compression for venous insufficiency, antibiotics for planktonic bacteria and debridement for necrotic tissue, each targeting a piece of a self-perpetuating triad. This section discusses diagnostic gaps that may perpetuate treatment failure, emerging innovations that promise improved outcomes, and potential integrated approaches that, if validated, could help break the cycle of chronicity [17,19,20,30].

Conventional culture-based methods, the long-time “gold standard” for identifying wound pathogens, may not adequately capture the biology of chronic wounds. These techniques are inherently biased towards the detection of actively dividing planktonic bacteria, a physiological state long since abandoned by biofilm-embedded organisms. Bacteria in the protective EPS matrix show reduced metabolic activity, altered gene expression and can enter a viable but nonculturable (VBNC) state. In this latent state, they maintain cellular integrity and virulence potential but resist cultivation on standard agar plates, giving false-negative results that routinely inform clinical decisions. This limitation is not trivial; standard culture fails to identify the most abundant microbial species in chronic infections in up to 70% of cases and in biofilm-associated conditions such as periprosthetic joint infections, molecular techniques detect pathogens in 80–100% of cases where traditional culture yields only 20–30% positivity [17,19].

The advent of molecular diagnostics is beginning to address these shortcomings by offering culture-independent strategies to directly detect bacterial DNA or RNA from clinical specimens. Next-generation sequencing (NGS), and in particular shotgun metagenomic sequencing, allows for a complete characterization of the entire polymicrobial community, including those anaerobic organisms that are generally not cultured. Metagenomic NGS has been particularly useful in overcoming diagnostic barriers related to biofilms, enabling early targeted therapy and potentially minimizing unnecessary exposure to broad-spectrum antibiotics. Complementary approaches such as peptide nucleic acid fluorescence in situ hybridization (PNA-FISH) allow rapid, species-specific detection of pathogens directly in biofilm matrices without the need for culture. Lab-on-a-chip technologies further extend the reach of diagnostics by integrating multiple assays on a single low-cost platform for point-of-care use in chronic wound management. Advanced technologies allow for deeper insights into biofilm dynamics, antimicrobial resistance gene expression and host–pathogen interactions beyond pathogen identification. These approaches could ultimately be used to predict disease risk, monitor disease progression, and personalize treatment plans, although clinical utility remains to be demonstrated. The application of artificial intelligence together with molecular datasets enhances pattern recognition and risk prediction, which may support precision medicine approaches for VLU care [17,20].

New evidence suggests a possible clinical utility of cytokine and homocysteine panels in identifying infected VLUs at high risk. Hyperhomocysteinemia is significantly more prevalent in infected VLUs (58.9%) than in uninfected ulcers (15.6%). Infected VLUs also have elevated levels of IL-6, IL-17A, and TNF-α in wound fluid. Validated, these biomarkers may represent accessible markers of risk of infection and may complement molecular diagnostics to inform clinical decision-making [29,34,36].

In vivo models of biofilm-associated infections provide an important translational link to study host–pathogen interactions in the complicated milieu of the wound microenvironment that cannot be achieved in vitro. These models permit the evaluation of biofilm formation, immune evasion strategies and therapeutic efficacy in conditions that better mimic human disease, which is important for the development and validation of novel interventions prior to clinical implementation [27,38,39].

Clinical management of VLU should be based on evidence-based interventions. Duplex ultrasound-guided assessment remains essential to identify the anatomical and hemodynamic basis of venous insufficiency. Early endovenous ablation of superficial venous reflux significantly accelerates ulcer healing and reduces recurrence at 3 years when combined with compression therapy. Multidisciplinary wound care teams, including vascular specialists, wound care nurses, and infectious disease consultants, are associated with improved healing rates and reduced recurrence. Systemic antibiotics should be reserved for clinical signs of spreading infection (cellulitis, lymphangitis, systemic symptoms), as routine use in chronic wounds associated with biofilm is not supported by current guidelines and may promote antimicrobial resistance.

### Limitations and Barriers to Clinical Implementation of Molecular Diagnostics

Despite their promise, there are several significant barriers that limit the current clinical utility of molecular diagnostic approaches for the management of VLU. There are no standardized protocols for collection, processing, and interpretation of sample data.

Differences in sampling technique, sample storage, and DNA extraction methods could have a significant impact on outcomes, and there is an urgent need for consensus guidelines. It is difficult to distinguish between colonization and actual infection.

Molecular techniques detect bacterial DNA from both viable and nonviable organisms, and the presence of microbial DNA does not necessarily indicate a clinically significant infection requiring antimicrobial intervention.

The high costs of NGS and other advanced platforms prevent their routine use in many clinical settings, especially in resource-limited settings. There is a lack of prospective studies showing that molecularly guided therapy improves clinical outcomes, particularly cure rates, time to cure, or prevention of recurrence, compared with conventional culture-based approaches.

Integrating molecular data into clinical decision-making requires specialized expertise that is not widely available, and the clinical significance of identified organisms, particularly anaerobes and organisms considered commensals, is often uncertain.

Finally, bioinformatic analysis of NGS data lacks standardization, and there are no universally accepted thresholds for defining “significant” microbial abundance or diversity. Until these limitations are addressed through collaborative efforts to establish guidelines, validate clinical utility, and reduce costs, molecular diagnostics should be considered a complement to, rather than a replacement for, conventional culture and clinical evaluation.

## 7. Therapeutic Strategies

**Figure 4:**
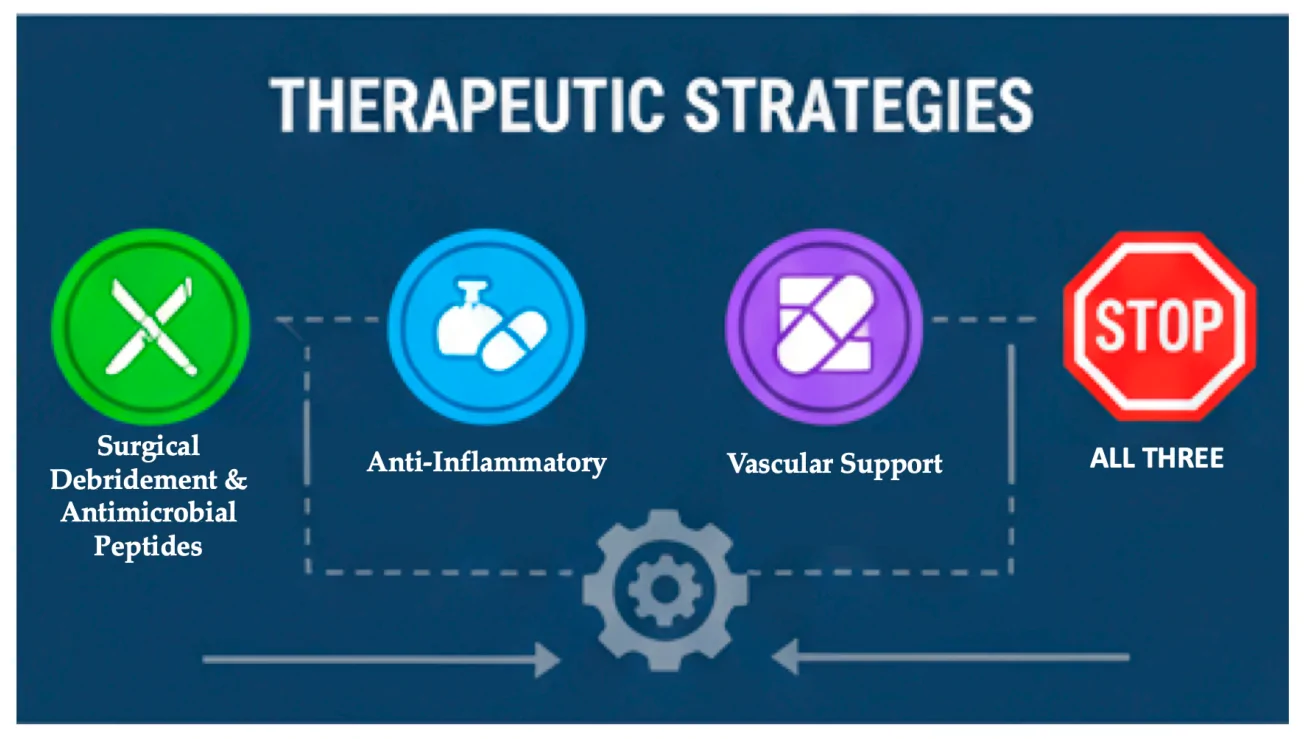
Multi-targeted intervention to break the vicious cycle.

Effective management of VLUs may require an integrated approach that simultaneously targets biofilm persistence, dysregulated inflammation and microvascular dysfunction, a hypothesis that requires clinical validation [17,19,30]. The main therapeutic goal, according to our proposed framework, would be to simultaneously address all three elements to potentially interrupt the self-sustaining loop, although this remains to be proven clinically (Figure 4).

Mechanical debridement continues to be the important first step to remove biofilm-contaminated tissue and expose the wound bed to topical therapies [10,28,40,41]. Disrupting the extracellular polymeric substance matrix that targets biofilms with enzymes has shown promise, as has antimicrobial peptides such as LL-37 which reduces biofilm density by up to 60% [42]. Bacteriophage therapy: a new strategy for biofilm-specific bacterial clearance [17]. Nanotechnology-based approaches, such as silver nanoparticles, metal-based nanoenzymes, and microneedle patches delivering cerium/zinc composites, have shown preclinical promise for targeting biofilm disruption with reduced systemic toxicity, although clinical evidence remains limited [20,42]. Povidone-iodine has broad-spectrum activity, good tolerability and ability to promote growth factors. Polyhexanide and octenidine are good alternatives. Management of the chronic inflammatory state involves approaches to decrease excessive cytokine load [41,42].

The local anti-inflammatory effects of immunomodulatory dressings containing antimicrobial peptides with dual antimicrobial and immunomodulatory effects. Systemic measures include optimisation of the management of comorbid conditions, especially diabetes, hypertension and hyperhomocysteinemia, through proper pharmacotherapy, nutritional supplementation and modification of lifestyle [10,25,26,32,41].

Compression therapy remains the standard of care for treating underlying venous insufficiency [18,30]. However, compression alone may be insufficient in the presence of biofilm or uncontrolled inflammation, a hypothesis that requires prospective evaluation [17,19,29]. Addressing hyperhomocysteinemia with folate, vitamin B12, and vitamin B6 supplementation may reduce vascular risk. Negative pressure wound therapy improves perfusion and mechanically debrides biofilm, while advanced biomaterials and smart dressings maintain moist wound healing and release antimicrobial agents in a controlled manner [27,37,38,39,43].

The integrated strategy, summarized in Table 3, emphasizes the coordinated management of biofilm, inflammation, and microvascular insufficiency.

Comprehensive wound assessment should include clinical signs of infection combined with advanced diagnostic techniques as appropriate. This includes the evaluation of wound size, depth and exudate, such as clinical signs of biofilm (delayed healing, foul odour, exudate), measurement of local and systemic inflammatory markers (IL-6, TNF-α, homocysteine) and molecular diagnostics (NGS, PNA-FISH) when biofilm is suspected. Compression therapy continues to be the mainstay of treatment for venous insufficiency, the initiator of the pathogenic cascade [17,19,20,33].

Compression optimization should be combined with vascular assessment, including ankle–brachial pressure index and duplex ultrasound, to ensure adequate venous return and maintain arterial sufficiency [30,43]. Management of biofilm should be performed with a multifaceted approach including precise or enzymatic debridement to physically remove biofilm, biofilm-disrupting agents targeting EPS matrix components, specific antiseptics (povidone-iodine, polyhexanide, octenidine) selected according to the wound type and biofilm characteristics and consideration of emerging therapies, such as antimicrobial peptides or bacteriophages, for refractory cases [10,17,28,40,41,42,44].

Objective measures of response to treatment should include wound size reduction (goal > 40% at 4 weeks), trends in inflammatory biomarkers (cytokines, homocysteine), clinical improvement (reduction in exudate, odour, pain), and assessment of biofilm burden (clinical signs, molecular diagnostics when available) [19,20,33].

Sustained healing needs addressing modifiable risk factors [25,26,32]. This includes optimizing diabetes control, hypertension management and nutritional support, weight management and smoking cessation, B vitamin supplementation for hyperhomocysteinemia, and education regarding adherence to compression therapy, wound care and recognition of signs of infection [19,33].

Although molecular diagnostics can identify biofilms with unparalleled accuracy, there are major barriers to their clinical application. Cost-effectiveness is questionable, as NGS costs between $500 and $1500 per sample, while standard culture costs between $50 and $100. It is unclear whether the improved diagnostic accuracy translates into improved clinical outcomes that justify the cost. The other barrier is access. NGS requires specialized equipment and bioinformatics skills that are available only at major academic institutions. Prospective validation in VLU populations is lacking. Most published studies have examined diabetic foot ulcers, pressure ulcers, or prosthetic joint infections, and sensitivity/specificity in wound exudate has not been rigorously established. Regulatory pathways are unclear, with the FDA approving only a limited number of molecular wound diagnostics. Most are laboratory-developed tests with variable quality control. The turnaround time (3–7 days for NGS versus 24–48 h for culture) can delay clinical decision-making, and the clinical significance of the microbes detected is not always interpretable. Finally, therapeutic options for the identified organisms may not be available, even if bacteriophages prove effective, and phage therapy is not widely available. These limitations should temper enthusiasm for molecular diagnostics and guide realistic implementation strategies.

## 8. Conclusions and Future Directions

We hypothesize that VLUs may result from pathogenic interactions between biofilm persistence, chronic inflammation, and microvascular insufficiency, extending beyond venous hypertension. Although each component is well supported by evidence, such as the presence of biofilm and delayed healing, dysregulated inflammation with elevated cytokines and impaired macrophage polarization, and microvascular dysfunction with aberrant angiogenesis, leukocyte recruitment, and fibrin deposition, the framework itself represents a hypothesis that requires empirical validation. The temporal and causal relationships between the components and the molecular links are still under investigation. The recommendation for simultaneous multi-targeted therapy is mechanistically inferred from this model but is based on preclinical evidence and plausibility rather than robust data from clinical trials. Novel combination strategies may prove beneficial but require validation in well-designed clinical trials. However, well-conducted randomized trials will be needed to demonstrate superiority over standard treatment and to determine the best combinations, sequencing, and timing of administration.

Prospective studies are needed to characterize the evolution of biofilm, inflammatory markers, and microvascular changes during healing. Such studies are important to establish temporal relationships, identify critical windows for intervention, and validate biomarkers predictive of healing or treatment failure. Transcriptomic, metabolomic, and proteomic approaches can reveal how biofilm-associated bacteria alter host inflammatory pathways and how chronic inflammation selects for virulent phenotypes. These approaches may also help explain how host responses paradoxically enhance biofilm persistence, thereby indicating novel therapeutic targets.

## Figures and Tables

**Table 1:** Biofilm Antimicrobial Resistance Mechanisms.

Mechanism	Principal Characteristic	References
EPS Diffusion Barrier	Positively charged antibiotics (e.g., aminoglycosides) bind to negatively charged matrix components	[17]
Metabolic Heterogeneity	Deeper cells exhibit reduced metabolic activity and slow growth	[17,19,27]
Reduced Antibiotic Susceptibility	Beta-lactams ineffective against dormant cells	[17]
Persisted Cells	Phenotypic tolerance: reversible, non-heritable, transient state	[17,19]
Genetic Resistance	Contrast with persistence: heritable vs. reversible	[19,24]
Horizontal Gene Transfer	Accelerates spread of resistance determinants within biofilm community	[18,19,24]
Treatment Complication	Biofilm bacteria survive therapy and repopulate upon treatment cessation	[17,18,19]

**Table 2:** The Host–Microbiome Interface in VLUs.

Direction	Factor	Mechanism	Clinical Consequences
Host → Microbiome	Age > 65 years	Immunosenescence; impaired immune surveillance [25,26,32]	Increased infection susceptibility
	Comorbidities (hypertension, diabetes, ischemic cardiomyopathy) Local immune dysfunction	[25,26,32] Systemic vascular and metabolic dysfunction; impaired perfusion [29,30,31,32] Impaired neutrophil chemotaxis/phagocytosis; M1 macrophage polarization [17,25,26,31]	Compromised host defense; poor healing Persistent inflammation; tissue destruction
Microbiome → Host	Contextual pathogenicity	Virulence is context-dependent, influenced by host microenvironment [31,36]	Commensals (e.g., *S. epidermidis*) become pathogenic under chronic wound conditions
	*S. epidermidis*	Biofilm-dependent pro-inflammatory cytokine induction [17,36]	Impaired re-epithelialization
	*P. aeruginosa* Functional selection	Degrades pro-inflammatory cytokines (G-CSF, GM-CSF, IFN-γ, IL-6, IL-12, IL-23, TNF-α) and chemokines (Gro-α, IL-8, IP-10, MCP-1, MIP-1α/β) [17,31] Chronic inflammation, oxidative stress, and antimicrobial exposure select for biofilm-forming, resistant phenotypes [17,24,31,36]	Suppression of classical inflammatory signs; bacterial persistence despite high burden Transformation of commensals into “accidental pathogens” impairing healing

**Table 3:** Triad-oriented management strategy for VLUs.

Component	Diagnostic Strategy	Therapeutic Intervention
Biofilm	Clinical signs; molecular diagnostics (NGS, PNA-FISH); [19,20]	Debridement; biofilm-disrupting enzymes; [10,17,20,28,40,41,42,44] antimicrobial peptides; bacteriophages; antiseptics
Inflammation	Cytokine panels (IL-6, IL-17A, TNF-α); clinical signs; [25,26,29,30]	Immunomodulatory dressings; comorbidity optimization; statins; nutritional support [25,26,32,33,41]
Microvascular	Homocysteine; ankle-brachial index; duplex ultrasound; [29,33,43]	Compression therapy; B-vitamin supplementation; negative pressure therapy [18,33,43]

## Data Availability

No new data were created or analyzed in this study.

## Supplementary Materials

The following supporting information can be downloaded at https://www.mdpi.com/article/10.3390/medsci14040453/s1. Supplementary File S1: Search strategy.

## Abbreviations

The following abbreviations are used in this manuscript:

VLUs	Chronic venous leg ulcers
EPS	Extracellular polymeric substances
M1	Pro-inflammatory macrophages
M2	Pro-reparative macrophages
ROS	Reactive oxygen species
SASP	Senescence-associated secretory phenotype
NETs	Neutrophil extracellular traps
TIMPs	Tissue inhibitors of metalloproteinases
VEGF	Vascular endothelial growth factor
GSH	Glutathione
LasB	Elastase B
MMPs	Matrix metalloproteinases
ECM	Extracellular matrix
NGS	Next generation sequencing
PNA-FISH	Peptide nucleic acid fluorescence in situ hybridization
ICAM-1	Intercellular Adhesion Molecule-1
VCAM-1	Vascular Endothelial Growth Factor
