# Supplementary material for: The Vicious Cycle of Biofilm, Host Inflammation and Microvascular Insufficiency in Venous Leg Ulcers
Source: Med Sci (Basel). 2026 Aug 2;14(4):453. doi: 10.3390/medsci14040453 (PMC13515536; doi:10.3390/medsci14040453)
Supplement: Supplementary file 1 [file medsci-14-00453-s001.zip › medsci-4454641-supplementary.pdf]

Supplementary File S1

Keywords and Subject Headings Used During the Search

**Database**	**Search strategy**
PubMed accessed, April 2026	("biofilm"[tiab] OR "microbial"[tiab] OR "EPS"[tiab])<br>AND ("inflammation"[tiab] OR "inflammatory"[tiab] OR "immune"[tiab])<br>AND ("venous ulcer"[tiab] OR "venous leg ulcer"[tiab] OR "chronic wound"[tiab])<br>AND ("microvascular"[tiab] OR "endothelial"[tiab] OR "venous insufficiency"[tiab])<br>AND<br>"2016/01": "2026/04"
Scopus accessed, April 2026	TITLE-ABS-KEY(<br>(biofilm OR microbial OR EPS)<br>AND (inflammation OR inflammatory OR immune)<br>AND ("venous ulcer" OR "venous leg ulcer" OR "chronic wound")<br>AND (microvascular OR endothelial OR "venous insufficiency")<br>AND<br>PUBYEAR > 2016 AND PUBYEAR < 2026
Web Of Science accessed, April 2026	TS=((biofilm OR microbial OR EPS)<br>AND (inflammation OR inflammatory OR immune)<br>AND ("venous ulcer" OR "venous leg ulcer" OR "chronic wound")<br>AND (microvascular OR endothelial OR "venous insufficiency")<br>PY=(2016-2026)
